# Pilates and dance to patients with breast cancer undergoing treatment: study protocol for a randomized clinical trial – MoveMama study

**DOI:** 10.1186/s13063-019-3874-6

**Published:** 2020-01-07

**Authors:** Leonessa Boing, Tatiana do Bem Fretta, Melissa de Carvalho Souza Vieira, Gustavo Soares Pereira, Jéssica Moratelli, Fabiana Flores Sperandio, Anke Bergmann, Fatima Baptista, Mirella Dias, Adriana Coutinho de Azevedo Guimarães

**Affiliations:** 1 Santa Catarina State University, Florianópolis, Brazil; 2 National Cancer Institute, Rio de Janeiro, Brazil; 3 University of Lisbon, Lisbon, Portugal

**Keywords:** Breast neoplasm, Quality of life, Physical activity, Dance

## Abstract

**Background:**

Breast cancer is a global public health issue. The side effects of the clinical treatment can decrease the quality of life of these women. Therefore, a healthy lifestyle is essential to minimize the physical and psychological side effects of treatment. Physical activity has several benefits for women with breast cancer, and Pilates solo and belly dancing can be an enjoyable type of physical activity for women with breast cancer undergoing clinical treatment. The purpose of this study is to provide a Pilates solo and a belly dance protocol (three times per week/16 weeks) for women undergoing breast cancer treatment and compare its effectiveness with that in the control group.

**Methods:**

The participants will be allocated to either the intervention arm (Pilates solo or belly dance classes three times per week for 16 weeks) or a control group (receipt of a booklet on physical activity for patients with breast cancer and maintenance of habitual physical activity routine). The Pilates solo and belly dance classes will be divided into three stages: warmup and stretching, the main stage, and relaxation. Measurements of the study outcomes will take place at baseline; postintervention; and 6, 12, and 24 months after the end of the intervention (maintenance period). The data collection for both groups will occur with a paper questionnaire and tests covering general and clinical information. The primary outcome will be quality of life (EORT QLQ-C30 and EORT QLQ-BR23), and secondary outcomes will be physical aspects such as cardiorespiratory fitness (6-min walk test and cycle ergometer), lymphedema (sum of arm circumference), physical activity (IPAQ short version), disabilities of the arm (DASH), range of motion (goniometer test), muscular strength (dynamometer test) and flexibility (sit and reach test), and psychological aspects such as depressive symptoms (Beck Depression Inventory), body image (Body Image After Breast Cancer Questionnaire), self-esteem (Rosenberg), fatigue (FACT-F), pain (VAS), sexual function (FSFI), and sleep quality (Pittsburgh Sleep Quality Index).

**Discussion:**

In view of the high prevalence of breast cancer among women, the implementation of a specific protocol of Pilates solo and belly dancing for patients with breast cancer is important, considering the necessity to improve their physical and psychological quality of life. Pilates solo and belly dancing are two types of physical activity that involve mental and physical concentration, music, upper limb movements, femininity, and social involvement. An intervention with these two physical activities could offer options of supportive care to women with breast cancer undergoing treatment, with the aim being to improve physical and psychological quality of life.

**Trial registration:**

ClinicalTrials.gov, NCT03194997. Registration date 12 August 2017. Universal Trial Number (World Health Organization), U1111-1195-1623.

## Background

Cancer is a global public health issue [1, 2]. Among the different types of cancer, breast cancer specifically is the most common among women worldwide [1, 3]. In Brazil, breast cancer is also considered the most common type of cancer among women [4]. Following the many advances in breast cancer treatment, there has been an increase in the 5-year survival rate from 78% to 87% in Brazil [4].

Despite the increase in the survival rate, breast cancer is a significant event in the patient’s life because of the serious side effects of clinical treatment [5], which compromise the patient’s functional capacity [6] and directly affect quality of life [7]. Living with these symptoms can result in emotional and physical exhaustion for these women [8], so a healthy lifestyle that involves good nutrition and regular physical activity is essential to minimize the psychological and physical side effects of treatment [9].

Considering this context, physical activity after the diagnosis of breast cancer, besides being a protective factor, is also auxiliary to the clinical treatment, minimizing the collateral effects and improving patients’ recovery [5, 9]. The American College of Sports Medicine (ACSM) recommends at least 150 min of moderate physical activity or 75 min of vigorous activity per week for patients with cancer [10]. Resistance training twice per week is also recommended to improve general physical health [10, 11]. A meta-analysis of 33 clinical trials regarding the psychological and physical benefits of physical exercise in women after breast cancer recommended physical activity at all treatment stages [12].

Among all types of physical activity that can relate to the healthcare of women with breast cancer, exercise that involves mind and body, such as Pilates and dance, can be beneficial [13–15]. These two types of activities are pleasant activities [16] that can promote emotional connections [17] and can be considered a moderate physical activity according to the ACSM recommendations.

Pilates solo includes resistance and stretching exercises synchronized with breathing, and it respects the principles of control, precision, centering, fluidity of movement, and concentration [18]. It promotes physical benefits for patients regarding functional capacity and muscle strength [19], and most exercises are performed in a position of dorsal decubitus [20] with control of speed, precision, and movement quality promoting the relaxation of the body [21], aspects that are considered consequences of breast cancer treatments, and therefore their recovery becomes essential.

Dance accompanied by music promotes movements with awareness of the body’s rhythms [22]. Belly dance specifically is directed only for women and is considered a form of exercise that associates body and mind through body movements involving especially the upper limbs and performed to the sound of traditional Arabic music [23, 24]. Because it is a dance that involves worship of the earth and the woman’s uterus, as well as feminine sensuality [24, 25], it can act in the rescue of femininity, softness, and beauty, exploring self-confidence and self-esteem of patients [23]. It is a modality of upper limb movement by controlling the arms using veils, tambourines, and vessels, and in this way it can promote physical benefits, considering the consequences of surgery and treatment in these patients [26].

Both the Pilates solo and dance have been the target of studies investigating physical exercise in patients after the diagnosis of breast cancer. Clinical trials that address the Pilates solo method demonstrate its benefits in several aspects, such as improving quality of life, functional capacity, and depressive symptoms [27], as well as benefits for muscle strength, pain, and upper limb functionality, after 8 weeks of treatment [19]. Also, improvements have been noted in external rotation and shoulder abduction in patients subjected to axillary emptying [21] and in shoulder range of motion (ROM), quality of life, body image, and mood after 12 weeks of intervention [14]. None of these studies had published protocols in Pilates solo methods for women with breast cancer.

There are several studies in the literature involving the effects of dance in patients with breast cancer [15, 17, 22, 26, 28–34]. However, published protocols for this population have not been identified; only two of these studies are characterized as randomized controlled trials [22, 33]. The modalities investigated included specific dance therapy methods [15, 17, 28–30, 33], classical ballet and jazz [31], traditional Greek dance associated with muscular strength training of the upper limbs [22], and the practice of circular dance [32] and ballroom dance for couples [33]. Belly dance is also investigated as a pilot study of our research group, identifying benefits in depressive symptoms, fatigue, and quality of life of women with breast cancer while undergoing treatment and after the treatment stage [26].

Thus, it is important to implement a specific protocol of dance and Pilates solo for patients with breast cancer because it has already been positively correlated with the health of women after diagnosis. For this study, belly dance was chosen as the modality of dance included in the protocol, considering the necessity of preserving the femininity of women during the disease [35]. Belly dancing can also address the physical and psychological needs of patients. Furthermore, this type of dance is a form of physical activity that associates the body and mind through movement, particularly involving the upper limbs, to the sound of traditional Arabic music [23–25]. This type of dance can also enhance the emotional aspects of women after the diagnosis of breast cancer because this practice involves expressive movements that facilitate the preservation of femininity, softness, beauty, trust, and security [23]. The Pilates solo was chosen because it favors lymphatic and blood drainage, improves posture, intensifies flexibility, and increases ROM and muscular strength [36]. When breathing exercises are added, the proposed exercises stimulate the thoracic lymphatic system, and thus they can promote a reduction in lymphedema, which improves muscle function and consequently improves quality of life [37].

This study protocol describes a randomized controlled trial of Pilates solo and belly dance (three times per week) for women after the diagnosis of breast cancer and compare its effects with a control group without intervention. The hypothesis is that the Pilates solo and belly dance protocol will promote improvement in primary (quality of life) and secondary (psychological and physical) outcomes in women after the diagnosis of breast cancer, providing a beneficial activity option for women with breast cancer. Our second hypothesis is that Pilates will lead to better improvements in the physical variables and belly dance will improve psychological variables.

## Methods

### Study design

This is a single-center, prospective, three-arm randomized clinical trial designed to assess the effects of Pilates solo and belly dance among women undergoing clinical treatment of breast cancer on the primary outcome of quality of life and secondary outcomes of physical aspects, such as cardiorespiratory fitness, lymphedema, physical activity, disabilities of the arm, ROM, strength, and flexibility, and psychological aspects, such as depressive symptoms, body image, self-esteem, fatigue, pain, sexual function, and sleep quality. Participants will be randomized to either a Pilates solo intervention group, a belly dance intervention group, or the control group. This study is conducted according to the Standard Protocol Items: Recommendations for Interventional Trials (SPIRIT) 2013 checklist: recommended items to address in a clinical trial protocol and related documents (Additional file 1).

### Ethical approval

This study will be conducted in compliance with the Declaration of Helsinki (1975), and it was approved by the Committee of Ethics in Research with Human Beings (CEPSH) of the Santa Catarina State University (UDESC) and the Ethics Committee of the Oncology Research Center (CEPON) under protocol no. 2.073.549 on May 19, 2017. This trial is registered at ClinicalTrials.gov with identifier NCT03194997. Any important protocol deviations or modifications will be communicated to CEPSH and the Clinical Trials website to approval, except when necessary to eliminate apparent immediate hazard(s) to human subjects. Also, the trial participants, trial registries, and the journal to which the protocol is submitted will be informed.

### Participants

The study will be conducted in the city of Florianópolis – State of Santa Catarina, Brazil. The participants will be women diagnosed with breast cancer who will be undergoing treatment in the Oncology Research Center (CEPON) at the time of data collection. The group will receive an explanation of the stages of the study, and after they provide consent to participate, they will sign an informed consent form and then be provided with an initial paper questionnaire for data collection.

### Eligibility criteria

The inclusion criteria are women aged 18 years or older, clinical stage 0 to III breast cancer, receiving adjuvant treatment with hormone therapy in CEPON at any time of the treatment cycle, and receiving the release of the oncologist responsible for the practice of physical activity or the physical therapy sector of CEPON. Exclusion criteria include the diagnosis of some orthopedic or neurological limitation that prevents the practice of physical activity, such as Parkinson’s disease, Alzheimer’s disease, or use of a wheelchair.

### Sample size

**Fig. 1:**
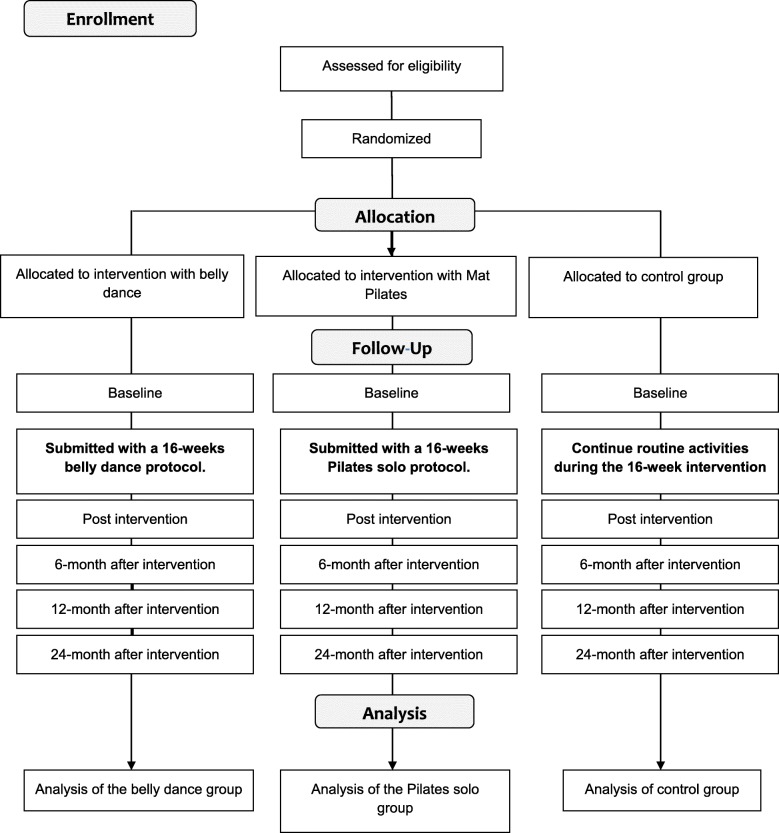
Flow diagram of the study participants according to Consolidated Standards of Reporting Trials (CONSORT 2010)

To calculate the sample size, the method of distinguishing between the means was used, being n = (α + β) 2 σ2/d2. The values of α = 0.05 and β = 0.80 were adhered to, for which, according to the Gaussian curve table, values of 1.64 and 0.84, respectively, were used. The difference between the means was obtained through the pilot study, and a variable considered for the calculation was quality of life, in which an average of the difference between all the scales in the before and after periods was found. This value of the expected selection was 6.15 ± 9.4. The expected variance (σ2) was 89.49. At the end of the analysis, after inclusion of a 30% margin of sample loss, a sample of 19 patients was selected for each group. Figure 1 shows the flow diagram of the study participants.

### Randomization and blinding process

The randomization of the sample will be performed by one of the researchers, who should have access to a list of patients with breast cancer (stages 0–III) who were receiving adjuvant treatment with hormone therapy at CEPON in the past 3 years, with the intention to achieve adequate participant enrollment to reach the target sample size. From this list, randomization will be carried out via a website (http://www.randomization.com), which will predict the allocation of patients in the three groups: group A, intervention with belly dance; group B, intervention with Pilates solo; and group C, control group that will be requested to maintain routine activities. The randomization will be stratified by age, dividing the patients between those younger and those older than 60 years of age, considering those older than 60 years of age, according to the Brazilian Statute of the Elderly, established by Law No. 10,741, dated October 1, 2003.

The data from the patients will be maintained only with the principal researcher to protect confidentiality before, during, and after the trial. Because the protocol is difficult to blind for the subjects and the instructors inasmuch as there is no proper way to perform sham physical exercise, all the data analysis will be performed by an external researcher. In this way, at least the data analysis will not be subject to interference.

### Pilates solo intervention

The women allocated to this group will participate in the Pilates solo protocol. The 16-week protocol will be implemented in 60-min Pilates solo classes three afternoons per week under supervision of an exercise science professional and a physiotherapist. The Pilates protocol will be divided into three stages:

1. *Warmup and stretching*: Including breathing, imprint and release, hip release, spinal rotation, cat stretch, hip rolls, scapula isolation, arm circles, head nods, and elevation and depression of scapulae exercises during warmup in all sessions.
2. *Main stage*: A brief explanation of the purpose of the class will be provided, and the exercises will take place as detailed in Table 1. To increase the load during the protocol, TheraBand and toning ball exercises will be added at the 10th session; in the 20th session, the exercise in the arms will be added, and from the 24th session, the exercise of spinal rotation will be realized with weight of 1 kg. Exercises will be performed according to each patient’s ability (principle of sports training) to avoid pain (during and after exercising) and embarrassment associated with possible physical or even psychological and emotional difficulties.
3. *Relaxation*: For this stage of the class, the patients will be invited to sit on the ball, spine stretch forward on the ball, self-stretch of cervical muscles on the ball (upper trapezius and scalene muscles), and actively mobilize the cervical spine. At the end of each class, a brief discussion will be held on the women’s perceptions regarding the objectives discussed in the beginning of class and whether they were achieved, and these data will be recorded by a third researcher to identify whether the participants enjoyed the class and felt that they achieved the objectives of the class.

**Table 1:** Sixteen-week belly dance protocol (48 sessions) for women after breast cancer diagnosis (Florianopolis SC, Brazil, 2018)

	Session 1 Slow songs (up to 80 bpm) Medium songs (up to 120 bpm) Fast songs (up to 150 bpm)	Session 2 Slow songs (up to 80 bpm) Medium songs (up to 120 bpm) Fast songs (up to 150 bpm)	Session 3 Slow songs (up to 80 bpm) Medium songs (up to 120 bpm) Fast songs (up to 150 bpm)
1	Belly dance presentation	Belly dance presentation	Belly dance presentation
2	Loosening of hips	Loosening of hips	Pendulum and side hit
3	Pendulum and side hit	Movements and steps sequence	Pendulum and side hit with displacement
4	Pendulum and side hit with displacement	Undulations and round	Movements and steps sequence
5	Undulations and round	Undulations and round with displacement	Movements and steps sequence
6	Undulations and round with displacement	Egyptian basics and twist	Movements and steps sequence
7	Egyptian basics and twist	Shimmys and variations	Shimmys and variations
8	Displacements, rotations, and use of space	Displacements, rotations, and use of space	Movements and steps sequence
9	Study of Arabic Dabke folklore	Study of Arabic Dabke folklore	Movements and steps sequence
10	Dance pitcher	Dance pitcher	Movements and steps sequence
11	Dance tambourine	Dance tambourine	Movements and steps sequence
12	Study of Arabic Khalije folklore	Study of Arabic Khalije folklore	Movements and steps sequence
13	Dance with the veil	Dance with the veil	Movements and steps sequence
14	Study of Arabic Dabke folklore	Dance tambourine	Study of Arabic Khalije folklore
15	Dance with the veil	Movements and steps sequence	Displacements, rotations, and use of space
16	Review of all the movements	Review of all the movements	Review of all the movements

*bpm* beats per minute

### Belly dance intervention

Women allocated to this group will participate in the belly dance protocol. The 16-week protocol will be implemented in 60-min belly dance classes three afternoons per week under supervision of an exercise science professional and a physiotherapist. The classes will be divided into three stages:

1. *Warmup and stretching*: The beginning of the class will include songs with up to 80 beats per minute (bpm), thus identified as slow pace. The sequence of movements for this class stage will cover large movements involving specific joints, including flexion, extension, abduction, adduction, and rotation, initiated by the upper body until it reaches the lower limbs, lasting 10 min.
2. *Main stage*: A brief explanation of the purpose of the class will be provided (i.e., the theory of dance or the specific step to be developed), followed by the practical part of the technical learning. The aim of this part will be for participants to learn the movements of the belly dance technique; to stimulate motor coordination, rhythm, and body awareness; and to improve aspects of flexibility and ROM of the upper limbs. The practice of the movements will be explored using individual, pair, or group dynamics involving movements corresponding to the rhythm of music or the rhythm stipulated for the women. The participants will have the artistic freedom to create their own pattern of movement based on the belly dance technique while respecting their own body awareness and allowing the expression of feelings. The evolution of the belly dance technique will be applied as outlined in Table 2. For this part of the classes, medium-paced music with up to 120 bpm will be used, as well as fast-paced music with up to 150 bpm. This part of the class will have an average duration of 40 min.
3. *Relaxation*: This stage will be developed from slow-moving practices with music up to 80 bpm, usually the same songs used in the initial warmup and stretching. With heart rate normalization, this part will last 10 min. At the end of each class, a brief discussion will be held on the women’s perceptions regarding the objectives discussed in the beginning of class and whether they were achieved, and these data will be recorded by a third researcher to identify whether the participants enjoyed the class and felt that they achieved the objectives of the class.

**Table 2:** Sixteen-week Pilates solo protocol (48 sessions) for women after breast cancer diagnosis (Florianopolis SC, Brazil, 2018)

Week	Session	Description of the activity
1	1	Explanatory session: What is Pilates, the basic principles of Joseph Pilates: breathing, centering, control, precision, fluency, and concentration. Clarify the positions: positioning of the pelvis, positioning of the rib cage, stabilization and movement of the shoulder girdle, and positioning of the head and cervical spine.
	2 and 3	Single-leg stretches, obliques (with feet flat on the floor), scissors, obliques roll, one-leg circle, side kick, preparation shoulder bridge performing simultaneously exercises of biceps with weight of 1 kg.
2	4	Single-leg stretches, obliques (with feet flat on the floor), scissors, obliques roll, one-leg circle, side kick, preparation shoulder bridge performing simultaneously exercises of biceps with weight of 1 kg, single-leg extension and roll up.
	5 and 6	Half-roll back (with over ball in the lumbar spine), roll up (pressing the magic circle with hands), single-leg stretch (with over ball between the shoulder blades), one-leg circle (with TheraBand around the thigh with both knees flexed), preparation shoulder bridge tightening the over ball between the knees and performing simultaneously exercises of biceps with weight of 1 kg, hell squeeze prone (pressing magic circle on ankles), side kick, spine twist.
3	7	Half-roll back (with over ball in the lumbar spine), roll up (pressing the magic circle with hands), single-leg stretch (with over ball between the shoulder blades), one-leg circle (with TheraBand around the thigh with both knees flexed), preparation shoulder bridge tightening the over ball between the knees and performing simultaneously exercises of biceps with weight of 1 kg, hell squeeze prone (pressing magic circle on ankles), side kick, spine twist.
	8 and 9	Half-roll back, single-leg stretches, double-leg stretches, preparation shoulder bridge (with feet on top of the ball), top-leg abduction, top-leg circles, staggered legs, both legs together.
4	10	Half-roll back, obliques (with feet on the floor, tightening the over ball, and extending the lower member to the side of the rotation of the trunk), double-leg stretch, scissors (with over ball in sacral region), preparation shoulder bridge (with feet on top of the ball), top-leg circles, both legs together.
	11 and 12	Obliques roll (with magic circle the knees), hundred (with feet on the floor), obliques (with feet on the floor and tightening the over ball between the knees, jackknife, both legs together and lateral flexion.
5	13	Obliques roll (tightening magic circle between knees), hundred (with feet on the floor), jackknife, top-leg abduction (with the lower limbs within the magic circle), top-leg circles, staggered legs (with the lower limbs within the magic circle).
	14 and 15	Roll up (with TheraBand on feet), slow double-leg stretch, double-leg stretch, preparation shoulder bridge (with feet on top of the ball), preparation shoulder bridge (with feet on top of the ball and performing extension and flexion of the knees), side kick (with ankle weights).
6	16	Hundred (with the feet on the floor), roll up (with TheraBand on feet), rolling like a ball, saw, preparation shoulder bridge (with feet on top of the ball), preparation shoulder bridge (with feet on top of the ball and performing extension and flexion the knees), double-leg kick.
	17 and 18	Single-leg stretches, obliques, roll over, preparation shoulder bridge performing simultaneously exercises of biceps with weight of 1 kg, hell squeeze prone (with magic circle on ankles), and spine stretch forward with pressing the magic circle with hands.
7	19	Hundred (with feet on the floor and tightening magic circle between knees), single-leg stretches, roll over, preparation shoulder bridge performing simultaneously exercises of biceps with weight of 1 kg, shoulder bridge and spine stretch forward with pressing the magic circle with hands.
	20 and 21	Side bend with knees supported, hundred (with hip and knees flexed 90 degrees), scissors with over ball in the sacral region, obliques roll (with weight of 1 kg), sing leg extension (with 1 kg ankle weights), jackknife.
8	22	Hundred (with hip and knees flexed 90 degrees), obliques roll (with weight of 1 kg), lateral flexion, single-leg extension (with 1-kg ankle weights), jackknife and hip twist with supported ribs.
	23 and 24	Roll up tightening magic circle, hundred (with hip and knees flexed 90 degrees), obliques, slow double-leg stretch, preparation shoulder bridge performing simultaneously exercises of biceps with weight of 1 kg, one-leg kick with 1-kg ankle weights, breaststroke with weight of 1 kg, side kick with 1-kg ankle weights and lateral flexion.
9	25	Roll up tightening magic circle, hundred (with hip and knees flexed 90 degrees), roll up with weight of 1 kg, slow double-leg stretch, roll over, preparation shoulder bridge performing simultaneously exercises of biceps with weight of 2 kg, one-leg kick with 1-kg ankle weights, side kick with 1-kg ankle weights and lateral flexion.
	26 and 27	Hundred (with hip and knees flexed 90 degrees and magic circle on our ankles), double-leg stretch, open-leg rocker, scissors in air, teaser (with feet flat on the floor), seal, preparation shoulder bridge with feet on top of the ball and performing simultaneously exercises of biceps with weight of 1 kg and spine stretch forward.
10	28	Hundred (with hip and knees flexed 90 degrees and magic circle on ankles), double-leg stretch, scissors in air, teaser (with feet flat on the floor) swimming, hip twist (with the ball between the ankles performing knee flexion and extension), side-kick kneeling, preparation shoulder bridge with feet on top of the ball and performing simultaneously exercises of biceps with weight of 1 kg, twist with knees on the floor, spine stretch forward.
	29 and 30	Hundred, side bend, obliques roll with weight of 1 kg, side bend, spine twist, swimming with weight of 1 kg, top-leg abduction with 1-kg ankle weights, top-leg circles with 1-kg ankle weights.
11	31	Hundred, side bend, scissors with over ball in sacral region, side bend, spine twist, swimming with weight of 1 kg, top-leg abduction with 1-kg ankle weights, top-leg circles with 1-kg ankle weights.
	32 and 33	Half-roll back, roll up, obliques, bicycle in air, teaser (with feet flat on the floor), side-kick kneeling with 1-kg ankle weights, one-leg circle with 1-kg ankle weights, staggered legs with 1-kg ankle weights, both legs together with 1-kg ankle weights and lateral flexion.
12	34	Shell stretch, half-roll back, roll up, obliques, teaser (with feet flat on the floor), one-leg circle 1-kg ankle weights, staggered legs 1-kg ankle weights both, legs together with 1-kg ankle weights and lateral flexion.
	35 and 36	Scissors in air, hundred (with magic circle on our ankles), single-leg stretch, teaser (with feet flat on the floor), rolling like a ball, swan dive (with hands supported without elevation of lower limbs), preparation shoulder (performing simultaneously exercises of biceps with weight of 1 kg), shoulder bridge and side kick with 1-kg ankle weights.
13	37	Teaser (with feet flat on the floor), hundred, double-leg stretches, open-leg rocker, swimming, side-kick kneeling, preparation shoulder bridge performing simultaneously exercises of biceps with weight of 1 kg, one-leg circle with 1-kg ankle weights, staggered legs with 1-kg ankle weights.
	38 and 39	Hundred, scissors with over ball in the sacral region, obliques roll (with weight of 1 kg), swimming, lateral flexion, single-leg extension with 1-kg ankle weights, side kick with 1-kg ankle weights, shoulder bridge.
14	40	Hundred, single-leg stretches, obliques, roll over, preparation shoulder bridge performing simultaneously exercises of biceps with weight of 1 kg, shoulder bridge, hell squeeze prone (with magic circle on our ankles), spine stretch forward (with hands on top of the magic circle performing a pressure), and swimming.
	41 and 42	Half-roll back, roll up, obliques, rolling like a ball, bicycle in air, one-leg circle with 1-kg ankle weights, staggered legs with 1-kg ankle weights, both legs together with 1-kg ankle weights and shoulder bridge.
15	43	Hundred, roll up, slow double-leg stretch, double-leg stretch, breaststroke, saw, preparation shoulder bridge (with feet on top of the ball), preparation shoulder bridge (with the feet on the ball performing extension and flexion of the knees), and double-leg kick with 1-kg ankle weights.
	44 and 45	Hundred, side bend, scissors with over ball in the sacral region, obliques roll with weight of 1 kg, preparation shoulder bridge with the feet on the ball performing simultaneously exercises of biceps with weight of 1 kg, top-leg abduction with 1-kg ankle weights, and top-leg circles with 1-kg ankle weights.
16	46	Scissors in air, teaser (with feet flat on the floor), hundred (with magic circle on our ankles), single-leg stretch, bicycle in air, rolling like a ball, swan dive with the hands supported without elevation of lower limbs, one-leg circle with 1-kg ankle weights, staggered legs with 1-kg ankle weights, preparation shoulder bridge with the feet on the ball performing simultaneously exercises of biceps with weight of 1 kg.
	47 and 48	Hundred, double-leg stretches, breaststroke, rolling like a ball, saw, preparation shoulder bridge (with feet on top of the ball), preparation shoulder bridge (with the feet on the ball performing simultaneously exercises of biceps with weight of 1 kg), and single-leg stretch with 1-kg ankle weights.

Warmup - classes 2 to 7 - breaststroke preparation (hands by hips) and preparation abdomen; classes 8 to 22/38 to 40/43 and 47 - breaststroke preparation (hands by shoulders) and preparation abdomen; classes 23 to 37/41 and 42/44 to 46 - breaststroke preparation (hands under forehead) and preparation abdomen; class 48 - breaststroke preparation (hands by shoulders) and preparation abdomen (with over ball between the knees)

Verification of the songs’ rhythm was performed by measuring the beats per minute according to the ballroom dance protocol used in the study by Braga et al. [38]. The songs will be categorized into the following groups: slow (up to 80 bpm), medium (up to 120 bpm), and fast (up to 150 bpm). The performance score was calculated using the BPM Detector Pro application.

### Safety and intensity

To control the intensity of the Pilates methods protocol and belly dance protocol, assuring that all the patients experience the same intensity of the intervention and to promote the safety of the practice of physical activity in these patients with breast cancer, heart rate (HR) control will be performed in every session using a Polar ProTrainer 5 (Polar Electro, Bethpage, NY, USA). HR values will be checked in four moments of the class: after the beginning of the class, after the warmup and stretching, after the main stage, and at the end of the class.

The safety of the intervention will be assessed every session, according to HR and patient self-report. If participants experience an adverse event, this will be brought immediately to the attention of the researchers. Adverse events will be evaluated by the researchers, who will make the decision to stop the study early, and researchers will take responsibility and provide all care to patients included in the study.

### Control group

Women allocated to this group will be asked to continue their routine activities during the 16-week intervention period. They will be contacted every 2 months by telephone. The intervention will be offered to this group in three meetings during the 16 weeks of the intervention. The first meeting will focus on stretching exercise to be performed at home on a regular basis, a second meeting will be about self-esteem, and the last meeting will be about prevention of lymphedema. These meetings will occur with the purpose of promoting an environment where these women can talk and share their experiences with other women with breast cancer and make sure that they also receive health education information, because they will not receive the exercise intervention in the first phase of the study. These meetings were a requirement of the Ethics Committee of CEPON, a hospital that will take part in the study in Brazil, to ensure that the control group also receives possible benefits of the study. Likewise, this strategy can improve the adherence of the control group because they will feel like a group and create social bonds.

Both groups, the experimental (Pilates solo and belly dance) and control groups, will receive, after 16 weeks of the intervention, an explanatory booklet on the benefits of practicing physical activity after breast cancer diagnosis, as well as instruction on the prevention of lymphedema. As a strategy to improve adherence of the subjects in the trials, all of the patients will be invited to social meetings, social media groups, and thematic classes according to specific calendar dates (e.g., Carnival, Easter, Halloween, Christmas), and they will receive a T-shirt from the project at the first meeting. Additionally, direct contact with the subjects who miss a class will occur via text messages and phone calls. These activities are planned to make the subjects feel familiar with the trial environment. For the 2-year follow-up, the intervention and control groups will be invited to participate in a physical activity program organized by the university. They will also be contacted through social media and text message once per month to motivate them to practice physical activity and remind them of future data collection. After the end of the study, besides publication in academic journals, the main results will be presented at the hospital in Brazil and shared with the patients in brochure format. Figure 1 presents the participant selection process and the execution of the steps of the protocol.

### Assessment

#### Primary outcome measure

The main aim of the study is to evaluate the impact of a 16-week Pilates solo and belly dance protocol on quality of life (≥ 2% of the baseline scores) in patients with breast cancer undergoing clinical treatment. Quality of life is the primary outcome at all time points of the study, namely baseline; postintervention; and 6, 12, and 24 months after intervention. Quality of life will be the primary outcome because it involves all the physical and psychological aspects of the life of women with breast cancer. Therefore, the maintenance collection will take place considering the modification in quality of life and other outcomes that the Pilates solo and belly dance intervention can promote in these women.

The primary outcome of quality of life will be investigated by using the European Organization for Research and Treatment of Cancer Quality of Life Questionnaire C30 (EORTC QLQ-C30) [39]. This instrument was created by the European Organization for Research and Treatment of Cancer (EORTC) in 1986, and this is the third version. Validated for the cancer population by Michels et al. [40], with Cronbach’s α values of 0.72 for global health, 0.86 for the functional scale, and 0.81 for the symptomatic scale.

The instrument consists of 30 questions, being multidimensional and self-applied. Its objective is to evaluate the quality of life in patients with cancer in the last 4 weeks. It presents five functional scales (physical, functional, emotional, social, and cognitive), a scale on global health status, three symptom scales (fatigue, pain, and nausea/vomiting), and six additional symptom items (dyspnea, appetite, constipation, diarrhea, and financial difficulties). The answers are presented in the form of a Likert scale following the classification 1 = not at all, 2 = a little, 3 = quite a bit, and 4 = very much. The only exception applies to the global health scale. This is composed of two questions that ask the patient to rate both health and quality of life in the last week according to grades of 1 to 7. In this case, 1 would be a poor quality of life and 7 a good quality of life.

The EORTC QLQ-C30 is complemented by specific cancer modules. In this study, the breast cancer module, QLQ BR-23, validated for the Portuguese language by Michels et al. [40], with Cronbach’s α values of 0.78 and 0.83, will be used. The QLQ BR-23 consists of 23 questions incorporated into multi-item scales. It measures the functional scales (body image, sexual functioning, sexual enjoyment, and future perspective) and symptom scales (systemic therapy side effects, arm symptoms, breast symptoms, and upset by hair loss). Classifications are also in the range of 0 to 100, where values ​​closer to 100 for the functional scale indicate better patient functionality and values ​​closer to 100 for the symptom scales show higher presence of symptoms.

#### Other outcome measures

As secondary outcomes, we will evaluate the physical and psychological variables associated with the quality of life. The physical outcomes are cardiorespiratory fitness, functional capacity, lymphedema, disabilities of the arm, ROM, strength, flexibility, and physical activity. In addition, the psychological outcomes are depressive symptoms, pain, fatigue, body image, self-esteem, sexual function, and sleep quality.

#### Cardiorespiratory fitness

To assess cardiorespiratory fitness, a submaximal incremental exercise test (85% of maximum heart rate [HRmax]) will be performed using a cycle ergometer (Excalibur Sport; Lode B.V., Groningen, the Netherlands). The protocol will start with a power of 20 W, and every 3 min, 15 W will be added, until the patient reaches 85% of HRmax, which will be identified through the equation (207 − 0.7 × age) [41]. In the initial 3 min of the test, the patient will be asked to remain in a resting position, accommodated by the cycle ergometer, to identify the values ​​of resting heart rate and oxygen consumption. Patients will be asked to maintain the rotation per minute (RPM) of the cycle ergometer always above 60 RPM. Expiratory gases and flow volume will be collected during the test and analyzed by using a calibrated metabolic system (Quark CPET; COSMED, Rome, Italy) to provide measurements of oxygen consumption. The heart rate will be monitored by a POLAR mark frequency and will be observed within the first 3 min of the test and at the end of each minute of the 3-min test stages. Also, every 3 min, the patient will be questioned about her perception of the exercise through the Subjective Perception Scale of Effort–Borg Scale [42]. This scale ranges from 6 to 20 points, where the sixth position would be the perception of “very easy” and 20 “exhausting.”

The 6-min walk test measures the distance a person can travel on a flat, rigid surface in 6 min. Its main objective is to determine tolerance to exercise and oxygen saturation during submaximal exercise [43]. Patients are asked to walk at their own pace as fast as possible during the 6 min, being allowed to walk slowly, stop, and/or rest when necessary and return to walking when they feel ready.

#### Lymphedema

The evaluation of lymphedema will be performed by calculating the arm volume, performed by measuring the circumferences of both upper limbs at five points distributed along the arm and forearm: at 21 cm and 11.5 cm above the olecranon to 7.5 cm, 14 cm, and 24 cm below the olecranon. The circumference will be obtained with the patient sitting, keeping the arm in abduction, flexed forearm, and hand resting on the chest. These measures are used to calculate the approximate volume of the five truncated cones formed at the points of circumference measurements. The sum of these five parts gives the total limb volume [44].

#### Disabilities of the arm

Evaluated through the Disabilities of the Arm, Shoulder and Hand (DASH) scale developed by Hudak et al. [45] and translated and validated for Brazil by Orfale et al. [46]. This instrument was developed to assess the disability and symptoms of single or multiple upper limb disorders. It contains 30 questions involving activities of daily living; symptoms of pain, tingling, and stiffness; and questions about social factors, work, sleep, and self-confidence. It has been used in other studies on patients with breast cancer [19].

#### Range of motion

To verify the ROM, evaluations of flexion, abduction, and external rotation of the shoulder according to previous studies with patients with breast cancer will be carried out [47] using a digital goniometer (Baseline Absolute Axis 360 degrees; Fabrication Enterprises, White Plains, NY, USA). The protocol used by Marques [48] will be performed for ROM assessment. The shoulder flexion movement will be performed with the subject lying down, and the flexion movement will be performed with the palm facing medially parallel to the sagittal plane. The fixed arm of the goniometer will be placed along the axillary line of the trunk, and the movable arm will be placed on the lateral surface of the humerus body facing the lateral epicondyle. For abduction, the individual must be seated. When performing the abduction movement, the palm of the hand will face anteriorly parallel to the frontal plane; the fixed arm of the goniometer should lie on the axillary line and posterior to the trunk; and the movable arm should lie on the posterior surface of the arm of the individual. In order to perform the external rotation, the position of the individual and the goniometer are the same; the individual will lie in the dorsal position, and the shoulder will be in abduction of 90 degrees, with the elbow flexed at 90 degrees and the forearm in supination. The arm of the goniometer should lie in the olecranon and the movable arm over the posterior region of the forearm directed to the third finger of the hand.

#### Strength of the upper limb

The muscle strength of the upper limb in both arms will be measured by the Chatillon® portable digital dynamometer (Ametek, Horsham, PA, USA), which can measure overall appendicular muscle strength and all body segments [49]. This equipment provides the value of the peak isometric maximum force exerted by the evaluated segment, and for this it requires a generation of fast force that does not fatigue the muscle. The maximum force generated is registered in newtons. The muscle groups responsible for flexion, extension, abduction, adduction, and internal and external rotation of the shoulder will be evaluated. The dynamometer will be placed over the specific location, and patients will be asked to perform force against the equipment for up to 5 s. Each muscle group will be evaluated three times, and the mean value of these evaluations will be used [50], with a 30-s interval between the tests, and bilaterally. In all cases, the patients will be instructed before the start and during the repetitions on the specific position.

#### Flexibility

The “sit and reach” test allows assessment of the flexibility of the coxofemoral joint [51]. The sit and reach box should be supported on a wall, and for evaluation, the patient is asked to keep the knees extended, bare feet resting in the sit and reach box, and hands overlapped on the horizontal surface of the box. The exercise should be performed with an anterior flexion of the spine, keeping the head between the arms, without flexing the knees, revealing a pause in the moment it reaches the maximum of the dotted line. Three replicates are performed, and the best mark among the three is considered.

#### Physical activity

The physical activity level will be investigated through the International Physical Activity Questionnaire (IPAQ–short version) [52]. The Brazilian validation and reproducibility were performed by Matsudo et al. [53] and had significant Spearman correlation and high reproducibility (rho = 0.69–0.71; *P* < 0.01) and validity of 0.75 observed compared with the instrument Computer Science & Applications (CSA; ActiGraph, Pensacola, FL, USA). It consists of six items that seek to verify the number of times the subject has practiced at least 10 continuous minutes of walking, moderate and vigorous physical activity, in the last week, in diverse involvements, namely labor, domestic, leisure, recreational, and sports. After completing the questionnaire, the participants can be classified into categories of sedentary, insufficiently active, and very active. The IPAQ also addresses the sitting time of individuals on weekdays and on weekends. There are two specific questions that ask: (1) How much time in total do you spend sitting on a weekday? and (2) How much time in total do you spend seated during on a weekend day? Data are presented in minutes per week.

#### Depressive symptoms

Depressive symptoms are investigated using the Beck Depression Inventory (BDI), a self-report questionnaire originally developed by Beck et al. [54]. It was validated in Brazil [55] and factorially validated for patients with cancer, indicating a Cronbach’s α of 0.82 [56]. It contains 21 multiple-choice objective questions related to depressive symptoms: sadness, pessimism, feeling of failure, dissatisfaction, guilty feelings, punishment feelings, self-dislike, self-criticism, suicidal thoughts, crying, irritability, and withdrawal from family or friends [56]. Each question provides four response options ranging from 0 to 3. The sum of the scores of each question provides a total score, ranging from 0 to 63, and the closer to 63, the greater the presence of depressive symptoms, indicating a higher degree of depression, and the greater the proximity to 0, greater the absence of depressive symptoms.

#### Pain

A visual analogue scale (VAS) will be used to assess pain. A VAS is a one-dimensional measure for assessing pain intensity. Composed of a 10-cm line with anchors at both ends, at one end of the line is marked “no pain” and at the other “worst pain imaginable.” The magnitude of the pain is indicated by marking the line, and a ruler is used to quantify the measurement on a scale of 0–100 mm [57].

#### Fatigue

Fatigue will be investigated by using the Functional Assessment of Cancer Therapy–Fatigue instrument (FACT-F). Validated in Brazil [58], it shows internal consistency of 0.91 for fatigue and 0.92 for the total FACT-F and has a total Cronbach’s α of 0.92. It is a self-report instrument aimed at patients with cancer that includes 13 items related to the perception of fatigue. Individuals will be asked to respond to each item with a score of 0 to 4, where 0 = not at all, 1 = a little bit, 2 = somewhat, 3 = quite a bit, and 4 = very much. In the total score, the possible interval is between 0 and 52, with a higher score indicating a level of less perceived fatigue.

#### Body image

Addressed by the Body Image After Breast Cancer (BIBCQ) questionnaire originally developed in Canada [59], which was translated, validated, and culturally adapted in Brazil [60] with values of Cronbach’s α for detailed internal validity for each scale, namely vulnerability (0.77), body stigma (0.86), concerns about the body (0.83), transparency (0.80), concerns about the arm (0.67), and limitations (0.72). This instrument aims to evaluate the body image after the diagnosis of breast cancer and can provide information related to the perceptions and attitudes regarding the side effects of breast cancer in the life of these patients. The BIBCQ is a questionnaire considered self-applicable, in which each item can be answered using a Likert scale. It consists of 44 questions divided into 6 scales, namely vulnerability, body stigma, limitations, concerns about the body, transparency, and concerns about the arm. In the end, the higher the score reaches, more compromised is the patient’s body image.

#### Self-esteem

The Self-Esteem Scale (EAR) developed by Rosenberg [61] will be used. This scale was validated for the population with cancer [62] and in Brazil [63]. It also received a validation review [64] with Cronbach’s α of 0.90. It is a one-dimensional measure consisting of ten statements related to a set of feelings of self-esteem and self-acceptance that determine global self-esteem. The total scale score varies from 10 to 40 points, and the following are used for categorization: (1) satisfactory or high self-esteem, those with a score greater than 31 points; (2) mean self-esteem, total score between 21 and 30 points; and (3) unsatisfactory or low self-esteem, scores less than 20 points. It is understood in this way that the greater the value reached by the woman on the scale, the better her self-esteem.

#### Sexual function

Sexual function is evaluated by the Female Sexual Function Index (FSFI) with cross-cultural validation [65], revealing a Cronbach’s α of 0.96. It is also validated internationally for patients with breast cancer [66] with Cronbach’s α of 0.70. This questionnaire consists of 19 questions grouped into 6 areas: desire, excitement, lubrication, orgasm, satisfaction, and pain. The sexual function score at the end of the analysis can vary from 2 to 36 points, considering that the higher the score obtained, the better the sexual function of the woman.

#### Sleep quality

Sleep quality is evaluated by the Pittsburgh Sleep Quality Index, which is validated [67] with a Cronbach’s α of 0.76. This instrument is composed of seven sleep-related areas: subjective quality, latency, duration, habitual efficiency, disturbances, use of sleeping medication, and daytime sleepiness. Scores range from 0 to 21 and correspond to overall sleep quality. In the end, scores up to 5 determine a good sleep quality and scores greater than 5 distinguish poor sleep quality.

#### Descriptive and control variables

**Fig. 2:**
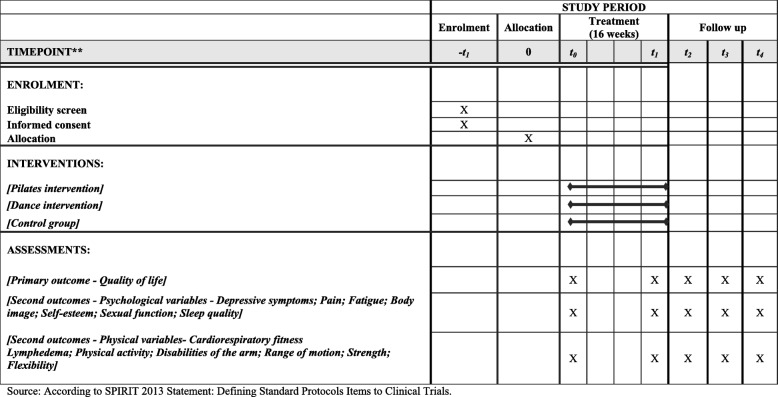
Template of recommended content for the schedule of enrollment, interventions, and assessments. *Source*: Standard Protocol Items: Recommendations for Interventional Trials (SPIRIT) 2013 statement: defining standard protocol items to clinical trials

The descriptive and control variables were divided into clinical variables (cancer stage, characteristics of treatment, previous clinical treatment, characteristics of surgical intervention, mammary reconstruction, date of surgery, presence of lymphedema, physiotherapy, and other diseases), sociodemographic variables (age, education, marital status, economic level, and occupation), and anthropometric measures (height and body mass). The descriptive and control variables will be acquired by self-report. The variables of the study regarding the Pilates solo and belly dance protocol are present in Table 3 and Fig. 2.

**Table 3:** Study assessments

Outcomes	Instruments	Visits					
		Baseline	Postintervention	3-month	6-month	12-month	24-month
Primary							
Quality of life	European Organization for Research and Treatment of Cancer Quality of Life Questionnaire C30 (EORTC QLQ-C30) and Specific for Breast Cancer (EORTC QLQ-BR23)	√	√	√	√	√	√
Other outcomes							
Psychological							
Depressive symptoms	Beck Depression Inventory (BDI)	√	√	√	√	√	√
Pain	Visual analogue scale (VAS)	√	√	√	√	√	√
Fatigue	Functional Assessment of Cancer Therapy–Fatigue (FACT-F)	√	√	√	√	√	√
Body image	Body Image After Breast Cancer (BIBCQ)	√	√	√	√	√	√
Self-esteem	Rosenberg Self-Esteem Scale	√	√	√	√	√	√
Sexual function	Female Sexual Function Index (FSFI)	√	√	√	√	√	√
Sleep quality	Pittsburgh Sleep Quality Index	√	√	√	√	√	√
Physical							
Cardiorespiratory fitness	Cycle ergometer 6-min walk test	√	√	√		√	√
Lymphedema	Sum of the arm circumference	√	√	√	√	√	√
Physical activity	International Physical Activity Questionnaire (IPAQ–short version)	√	√	√	√	√	√
Disabilities of the arm	Disabilities of the Arm, Shoulder and Hand (DASH)	√	√	√	√	√	√
Range of motion	Goniometer test	√	√	√	√	√	√
Strength	Dynamometer test	√	√	√	√	√	√
Flexibility	Sit and Reach Test	√	√	√	√	√	√

#### Data collection

Data will be collected using an interview format with a paper questionnaire and physical tests. The principal investigator of the study, who received previous training, will conduct a 50-min interview. The questionnaire will cover general and clinical information, quality of life, and psychological variables. The physical variables will be investigated by the specific tests. All data collection will be administered before the beginning of the intervention (baseline collection) and after the conclusion of the 16-week protocol (postintervention collection), as well as 6, 12, and 24 months after intervention (maintenance collection) (see Fig. 1). The maintenance collection will take place considering the health behavior change that the belly dance and Pilates solo intervention can promote in women with breast cancer.

For the control group, data collection will be conducted using the same paper-based questionnaire and tests applied to the intervention group, with data on general and clinical information, quality of life, and physical and psychological variables. The collection will be scheduled with the participants and will take place at the same intervals as for the experimental group, before the start of the intervention (baseline collection) and after conclusion (postintervention collection) by the same principal investigator, as well as 6, 12, and 24 months after the intervention (maintenance collection). All the data collection of intervention and control groups will occur at Santa Catarina State University.

Patients who discontinue in the intervention or control group who did not show for the meeting will be collected as well and analyzed as an intention-to-treat group. During the intervention and data collection, the researchers will collect spontaneous feedback from the patients to guarantee that the study will not have any adverse events. Data from all groups (experimental and control) will be held according to the principles of good clinical practice and the Declaration of Helsinki and will be treated with confidentiality, following the current privacy policy.

#### Statistical analysis

First, a spreadsheet will be created using the Excel 2016 software (Microsoft, Redmond, WA, USA), from which the data will be transferred to IBM SPSS Statistics version 20.0 software (IBM, Armonk, NY, USA) for analysis. Descriptive statistics (mean, standard deviation, and percentage) for the characteristics of the sample will be computed. To investigate the relationship between general and health information of the control and experimental groups, chi-square or Fisher’s exact tests will be used. To analyze differences in the experimental and control groups in the baseline, postintervention, and maintenance periods, two-way analysis of variance with repeated measures and Holm-Šídák comparison tests will be conducted. Confounder variables will be considered in the analyses as type of treatment, type of surgery, age, and weight status. The analysis will be performed according to the protocol and intention to treat, meaning that all the patients will be evaluated according to the randomization process. Missing data will be handled using the multiple imputation method. The significance level of 5% will be two-sided.

## Discussion

We present a 16-week Pilates solo and belly dance protocol for women after breast cancer diagnosis. In the literature, the benefits of physical activity for women with breast cancer are well established. Systematic reviews have reported improved quality of life and cardiorespiratory capacity and reduced fatigue after practice of physical exercise during breast cancer treatment [12, 68–70]. The proposal of this study is to present a protocol of Pilates solo and belly dance (three times per week) for women diagnosed with breast cancer and compare its effects with a group without intervention, considering that these are two kinds of activities that valorize mind and body and can bring different outcomes and benefits for women with breast cancer [71].

Dance can represent both psychotherapeutic treatment and a form of physical activity, based on body awareness, expression, and acceptance, to facilitate physical, emotional, cognitive, and spiritual integration [17]. Moreover, through the socialization context promoted by dance, benefits are revealed in relation to decreased feelings of loneliness and misunderstandings with others [30]. Pilates was created by Joseph Pilates as a method based on Eastern mind-body-spirit theories combined with Western theories, according to the following six principles: centralization, control, concentration, fluidity, breathing, and precision [71]. Its practice provides shoulder and pelvis stability and improves posture, stretching capacity, muscular strength, and mind-body connection [36].

In a systematic review of dance and breast cancer published by our group, we identified dance as a viable alternative of adjuvant treatment for patients who have passed through breast cancer, as well as claiming that it can promote psychological benefits and improve strength and ROM of the upper limbs [72]. In this scenario, studies involving dance and breast cancer may involve specific dance therapy methods [15, 17, 28–30, 33]; traditional dance techniques, such as classical ballet and jazz [31]; the practice of traditional Greek dance associated with the training of the upper limbs [22]; as well as circular dance [32] and ballroom dance [33]. However, none of these studies presented published protocols demonstrating the importance of a belly dance protocol for women after the diagnosis of breast cancer. Further publication of a belly dance protocol will improve the possibility of generalization of the study, assuring the external validity.

The use of the Pilates method in patients with breast cancer was evaluated by a systematic review of four studies, and it was determined that the method leads to an improvement in patients’ ROM, pain, and fatigue [73]. Other evidence related to the benefits of the Pilates method for the health of these women supported improved quality of life, reduced pain and fatigue, decreased lymphedema, and increased upper limb functionality [21, 27, 37, 74]. These studies were published on Pilates interventions, but there is no protocol study for women with breast cancer. Also, the further publication of a Pilates solo protocol will improve the possibility of generalization of the study, assuring the external validity.

Methods of dance therapy are generally similar, taking advantage of subjective approaches to the perceptions of body and movement fluency in relation to feelings [15, 17, 28–30, 33]. These methods can comprise the use of conscious walks and drives, verbal feedback, exploration of specific body parts, the use of different movement intensities (light and slow to energetic and active), and the work in pairs [28]. These studies have shown positive results in relation to psychological and physical aspects of women after the diagnosis of breast cancer. However, they do not include the validation of a protocol, which, therefore, does not allow the study to be replicated by other researchers and does not indicate the frequency, duration, or intensity of the movements, as well as the beats per minute of the music used.

The belly dance protocol presented in this study addresses a form of dance that has predetermined movements and specific techniques and was developed following a specific progression to the correct learning model. In this sense, belly dance has been chosen as the model for the intervention protocol for being an enjoyable practice that involves an intimate relation between movement and emotion. It also preserves the female identity and awakens a spontaneous body language, with beneficial movements that respect the individuality of each practitioner. Belly dance is also characterized as a practice that offers intense movement of the upper limbs [23–25], which directly benefits women, addressing limitations caused by the disease, such as the development of lymphedema and decrease of ROM. A pilot study was developed by the research group itself and has been shown to be an effective possibility for interventions with patients with breast cancer [26]. In the pilot study, the intervention was only performed for 12 weeks, often twice weekly and with 60 min of duration per session, but already demonstrated benefits in breast cancer related to quality of life, depressive symptoms, and fatigue. Also, the adherence was 78.6% (95% confidence interval, 71.3–85.9).

The Pilates intervention protocol presented here has not yet been performed in women with breast cancer, and it is of great relevance as an adjuvant therapy in the treatment of these women. The protocol was developed to achieve the great benefits reported in the international literature, including improvement in quality of life and reduction in the physical and psychological effects of adjuvant breast cancer treatment. In addition, this protocol influences and encourages the practice and maintenance of physical activity after treatment, because the practice of physical activity reduces the risk of breast cancer recurrence [75]. The exercises include stretching of the upper and lower limbs, upper limb mobility, and strengthening of the upper and lower limbs and abdomen, with consideration and respect for each patient’s limitations, and most exercises are performed in the supine position, avoiding impact to the joints.

The time of intervention of 16 weeks for this protocol was chosen considering the pilot study [26] and the systematic review of breast cancer and dance [72] and Pilates [73]. The pilot study of 12 weeks showed psychological benefits in women with breast cancer, and the classes were performed in 24 sessions. Therefore, to improve physical and psychological aspects in this protocol, it was decided to explore twice the number of sessions, leading to a 48-session protocol over 16 weeks. In the systematic review of dance and breast cancer, it was demonstrated that interventions were performed with a range of 3–24 weeks, with one to three sessions per week and 1–3 h per session. It was also observed that most of the studies identified in the systematic review about Pilates and breast cancer had their interventions with a total duration of 8 weeks, frequency of three times weekly, and sessions of 45–60 min [73]. Thus, as an average of these findings, we also propose 16 weeks with three 60-min sessions per week.

Due to the lack of a systematic and specific protocol for patients with breast cancer and the importance of acting with adjunctive treatment, a Pilates solo and belly dance intervention protocol was developed to improve quality of life, as well as to mitigate the psychological and physical outcomes of women after breast cancer diagnosis. With these being two kinds of physical activity that are known worldwide, there is the possibility of application in other locations. Finally, Pilates solo and belly dance are characterized as important physical activity options for this population that can minimize the side effects of the disease and its treatment, assisting in the patients’ recovery.

### Trial status

Protocol version 1, dated from May 17, 2019. The trial is ongoing and currently enrolling. It is expected to be ongoing from January 2019 to December 2020.

## Supplementary information

**Additional file 1.** SPIRIT 2013 checklist: Recommended items to address in a clinical trial protocol and related documents.

## Data Availability

Data are available from the authors upon request.
